# Principles of Functional Circuit Connectivity: Insights From Spontaneous Activity in the Zebrafish Optic Tectum

**DOI:** 10.3389/fncir.2018.00046

**Published:** 2018-06-21

**Authors:** Emiliano Marachlian, Lilach Avitan, Geoffrey J. Goodhill, Germán Sumbre

**Affiliations:** ^1^Institut de Biologie de l’Ecole Normale Supérieure (IBENS), Ecole Normale Supérieure, CNRS, INSERM, PSL Université Paris, Paris, France; ^2^Queensland Brain Institute, The University of Queensland, Brisbane, QLD, Australia; ^3^School of Mathematics and Physics, The University of Queensland, Brisbane, QLD, Australia

**Keywords:** zebrafish, optic tectum, spontaneous activity, functional connectivity, sensory experience, two-photon calcium imaging

## Abstract

The brain is continuously active, even in the absence of external stimulation. In the optic tectum of the zebrafish larva, this spontaneous activity is spatially organized and reflects the circuit’s functional connectivity. The structure of the spontaneous activity displayed patterns associated with aspects of the larva’s preferences when engaging in complex visuo-motor behaviors, suggesting that the tectal circuit is adapted for the circuit’s functional role in detecting visual cues and generating adequate motor behaviors. Further studies in sensory deprived larvae suggest that the basic structure of the functional connectivity patterns emerges even in the absence of retinal inputs, but that its fine structure is affected by visual experience.

## Introduction

Sensory brain areas are continuously active even in the absence of external stimulation. This ongoing spontaneous activity, defined as the intrinsic brain activity not driven by sensory stimuli, was once considered to be merely biophysical noise which interferes with brain computations (Tolhurst et al., 1983; Faisal et al., 2008). However, this view has changed in recent years, as spontaneous activity has been found to be structured in space and time (Kenet et al., 2003; Fiser et al., 2004; Luczak et al., 2007, 2009; Smith and Kohn, 2008; Ringach, 2009; Kirkby et al., 2013; Jetti et al., 2014; Romano et al., 2015). In sensory brain areas, spontaneous activity can exhibit spatial patterns that match functional sensory maps (Kenet et al., 2003; Jetti et al., 2014; Romano et al., 2015).

This ongoing spontaneous activity could reflect any or all of: (1) top-down signals associated with cognitive process (e.g., attention, memory, Engel et al., 2001; Harris and Thiele, 2011); (2) preparatory activity associated with self-generated behaviors (e.g., readiness potential; Libet et al., 1983); (3) a readout of the functional connectivity of neural circuits (which neurons are connected and the strength of these connections); and (4) a Bayesian prior for expected patterns of sensory activity (Berkes et al., 2011). Therefore, patterns of spontaneous activity may contain information about the circuit’s functional connectivity constraints and/or preferred states (Kenet et al., 2003; Miller et al., 2014; Romano et al., 2015).

**Figure 1:**
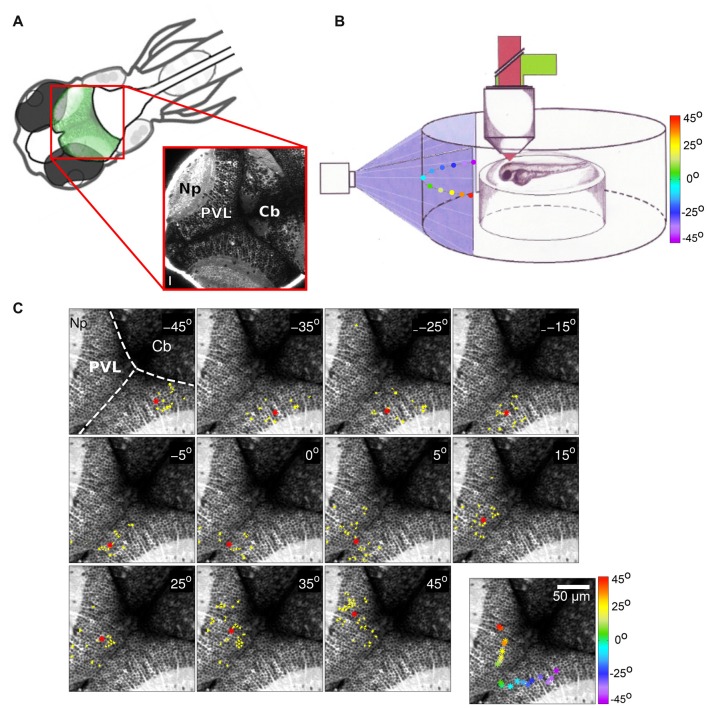
**(A)** Top left: schematic dorsal view of an 8-days-post-fertilization (dpf) zebrafish larva. The optic tectum is indicated in green. Bottom right: optical section of a Tg(huC:GCaMP5G) zebrafish larva showing pan-neuronal GCaMP5 expression corresponding to the area in the top left scheme (red rectangle). The image was obtained using a two-photon microscope. PVZ, Periventricular layer; Np, neuropil; Cb, cerebellum. Scalebar: 15 μm (reproduced with permission from Pietri et al., 2017). **(B)** Schematic drawing of an agarose-embedded zebrafish larvae in the recording chamber. The colored dots represent the different stimuli positions projected on a screen (color scale). **(C)** Examples of neuronal groups consistently activated by light spots at different positions in the field of view of the larva (azimuth angle, top right corner). Activated neurons are colored in yellow, and the neuronal groups’ centroids are depicted as red asterisks. Top left: anatomical landmarks. Dashed lines delineate the tectal-cerebellar and inter-hemispheric tectal boundaries. Cb, cerebellum; Np, tectal neuropil; PVL, periventricular layer. Bottom right panel: the centroids colored according to the azimuth angle (color bar on the right). The centroids evenly tile the contra-lateral rostro-caudal (reproduced with permission from Romano et al., 2015).

The zebrafish larva is an excellent model to study these principles of functional connectivity through the interrogation of the brain’s ongoing spontaneous activity. Its transparent skin, small size and cutaneous breathing, in combination with optogenetics and cutting-edge imaging approaches, such as 2-photon and SPIM, allow the dynamics of large neuronal populations to be monitored, with single-neuron resolution, in an intact, non-paralyzed, non-anesthetized behaving vertebrate (Figure 1A). From a behavioral point of view, upon hatching the larva needs to immediately catch prey and avoid predators in order to survive. These constraints force the larva to rapidly develop complex behaviors. However and despite their complexity, these behaviors are based on discrete bouts, thus facilitating their classification (Marques et al., 2018), analysis and correlation with different aspects of neuronal circuit dynamics.

In this review article, we discuss recent work analyzing spontaneous activity in the optic tectum (homologous to the superior colliculus in mammals) of the zebrafish larva, to shed light on its functional connectivity and the mechanisms underlying its emergence. Overall, these studies support the idea that spontaneous activity reveals important features of the functional role of neuronal circuits. If we consider the brain not just as a purely stimulus-driven data processor, but rather as an active system with rich intrinsic dynamics that interact with environmental sensory information, spontaneous activity could bias the response to sensory information, and thus confer a specific role for expectations in the animal’s ability to interpret and respond rapidly to relevant external stimuli.

## The Zebrafish Optic Tectum: A Model for Studying Functional Neuronal Circuitry

In zebrafish the optic tectum receives mainly sensory inputs from the retina, but also from other modalities (Thompson et al., 2016; Pietri et al., 2017). Its main functional role is to detect sensory stimuli, process them, and thus generate appropriate motor behaviors such as prey capture (Gahtan et al., 2005; Krauzlis et al., 2013). Tectal neurons receive inputs from retinal ganglion cells (RGCs), exclusively from the contralateral eye, that synapse on the tectal neuropil (Np) creating a functional retinotopic tectal map of the contralateral visual field (Burrill and Easter, 1994; Niell and Smith, 2005; Romano et al., 2015). The elevation of the contralateral visual hemifields are represented in the dorso-ventral tectal axis, whereas the azimuth of the hemifields are mapped along the rostro-caudal tectal axis.

Neurons in the optic tectum are selective for different stimulus features in the visual field such as size (Preuss et al., 2014; Barker and Baier, 2015), position (Niell and Smith, 2005; Romano et al., 2015; Figure 1B), or direction (Gabriel et al., 2012; Hunter et al., 2013; Romano et al., 2015). Responses to visual cues in the optic tectum take the form of co-active neuronal groups, i.e., a population code (Georgopoulos, 1988; Niell and Smith, 2005). Some stimulus features, for instance direction selectivity, are represented with no apparent tectal spatial organization (Hunter et al., 2013; Romano et al., 2015). However, other features, such as position in the visual field, are represented by compact topographically organized groups of neurons, indicating a rough preservation of spatial relationship between the retina and the tectum (Figure 1C). It is often assumed that this topography of wiring is essential for the animal to decode the position of a given target in the visual field. To examine this Avitan et al. (2016) used evoked tectal neural activity to decode the positions of small spots presented to the larva. While the performance of a topography-based decoder was significantly better than chance, it was still inferior to performance obtained using methods that do not rely on the spatial position of tectal neurons, in particular a linear decoder and a maximum likelihood decoder. A computational model of the zebrafish visual system reproduced these results and confirmed that topography-based decoding performs well only for widely separated stimuli. Theory shows that a topography-based decoder can perform as well as a maximum likelihood decoder only when the map is perfect, i.e., dense, regular with little background noise (Snippe, 1996). However, these conditions are usually violated in the tectum with only large-scale rough topography (Niell and Smith, 2005; Romano et al., 2015) and a substantial proportion of misplaced cells (Northmore, 2011). Thus while topography provides a convenient initial method for decoding, it could be replaced by more statistically sophisticated methods as the animal gains more experience in the world.

While structured activity of neuronal populations takes place in response to visual input, similar tectal activity is observed spontaneously in the absence of visual cues. The emergence of this activity may be guided by intrinsic retinal and/or tectal activity and visual experience. We now discuss recent studies which addressed these issues by recording spontaneous tectal activity over development, both in the presence and the absence of retinal input.

## Spontaneous Activity as a Proxy for the Circuit’s Functional Connectivity

**Figure 2:**
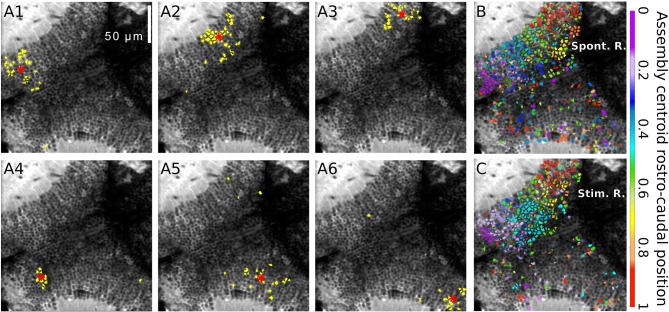
Spontaneously emerging neuronal assemblies. **(A1–6)** Topographies of six examples of spontaneous neuronal assemblies. The neurons belonging to each of the assemblies are labeled in yellow. Red asterisks: assembly centroids. **(B)** Example of the spontaneously emerging spatial organization where all the spontaneous assemblies whose centroids lay on the right tectum. Neurons belonging to assemblies with similar topographic centroids were similarly colored. The colors represent the average normalized rostro-caudal centroid position of their assemblies. **(C)** The same representation for the light-spot-induced responses. Note the resemblance between **(B,C)**, showing a graded change in colors along the rostro-caudal axes. Scalebars: 50 μm (reproduced with permission from Romano et al., 2015).

To learn about the functional connectivity of the optic tectum, Romano et al. (2015) monitored tectal spontaneous activity in the absence of any sensory stimulation. Neuronal populations displayed sparse basal activity, with low average neuronal activation frequencies (0.016 ± 0.015 Hz) and involving less than ~1% of the imaged population simultaneously active at any one time. Nevertheless, the pair-wise temporal correlations were significantly larger than those for null models generated by permutation of the identity of neurons in each experiment. Interestingly, these highly correlated neurons displayed a spatially organized structure (groups of neurons that are topographically compact). These functional neuronal assemblies emerged in the absence of sensory stimulation but were spatially organized similarly to the neuronal groups evoked by small light-spots presented at different positions of the field of view. They were composed of neurons which share similar spatial tuning curves, but with no specific similarity in terms of their direction selectivity (Romano et al., 2015; Figure 2).

Given the retinotopic organization of the retinal inputs to the tectal Np, it is possible that the assemblies are the result of spontaneous activity relayed from the retina. However, physical removal of the RGCs (enucleations) 1–3 days prior to the spontaneous recordings did not significantly affect the assemblies and their spatial organization. This suggests that spontaneous neuronal assemblies of the optic tectum are not the simple outcome of correlated feed-forward inputs arriving from the retina, but rather are generated intrinsically in the tectum. However, as discussed later, early enucleation does have an effect on the development of the tectum.

The spontaneous activations of the neuronal assemblies showed a facilitatory effect, where neurons were more likely to be activated when the spontaneous network activation better resembled the pattern of the assembly they belong to. Thus, the spontaneous neuronal assembly patterns represent network states that are “preferred” by the assembly neurons. This is similar to pattern completion in attractor-like circuits (Knierim and Zhang, 2012), and acts like a prior on the stimulus distribution. This could help the larva to better detect prey in low-contrast environments, and cope with the observed variance of the stimulus in the natural environment.

Furthermore, the spontaneous tectal activity patterns matched, with significant cellular precision, those induced by visual stimulation. More precisely, they were similar to neuronal responses induced by visual stimuli of a size matching that of the natural prey of the larva (e.g., Paramecia), rather than visual stimuli of larger sizes. This implies that the assemblies are highly similar to those evoked by the visual stimuli, supporting their biological relevance. This suggests that the tectal functional circuit connectivity is adapted to respond preferentially to visual stimuli of biological relevance for the survival of the animal (e.g., the larva’s prey), and therefore, that the tectal circuitry is adapted for its functional role (Romano et al., 2015).

Similarity between evoked and spontaneous patterns of neural activity has been observed in several species (Kenet et al., 2003; Berkes et al., 2011; Miller et al., 2014; Romano et al., 2015). While this similarity could also emerge due to circuitry constraints, it is much higher for behaviorally relevant stimuli than for non-behaviorally relevant ones (Berkes et al., 2011; Romano et al., 2015), suggesting that these adaptations reflect a general principle governing circuit organization.

## Emergence and Development of the Tectal Functional Circuitry

An important question in neuroscience is to understand the role of sensory experience and genetic factors in the development of neuronal circuits. To help address this, two recent articles (Avitan et al., 2017; Pietri et al., 2017) investigated the emergence of neural assemblies in the tectum during a critical developmental time window (4–9 days-post-fertilization, and 2.5–8 dpf, respectively). From 48 h post-fertilization RGCs begin to connect with the tectal Np. At 3 dpf the optokinetic response is already established, by 5 dpf larvae are capable of tracking and capturing prey, and by 8 dpf the optic tectum is structurally and functionally relatively mature (Niell and Smith, 2005). Pietri et al. (2017) reported that the frequency, the duration, the amplitude of calcium events of tectal neurons and the temporal correlations between them change abruptly between 2.5 dpf and 3 dpf, the period at which the retina establishes a functional connection with the optic tectum (Niell and Smith, 2005; Pietri et al., 2017).

In the zebrafish optic tectum, the size of the visual receptive fields increases from 4 dpf to 6 dpf and then refines to the same size as that at 4 dpf by 8–9 dpf (Zhang et al., 2011). The excitatory components of the receptive field play a more important role in determining this developmental profile than the inhibitory components. During this developmental period, GABAergic responses most likely switch from depolarizing to hyperpolarizing currents, and thus functional pruning of feedforward inputs is likely the most important factor in receptive field refinement (Zhang et al., 2011). When monitoring the spatial structure of the spontaneous activity, Pietri et al. (2017) found that the spatial organization of the neuronal assemblies followed similar dynamics as those observed for the development of the receptive fields of single neurons (Zhang et al., 2011).

**Figure 3:**
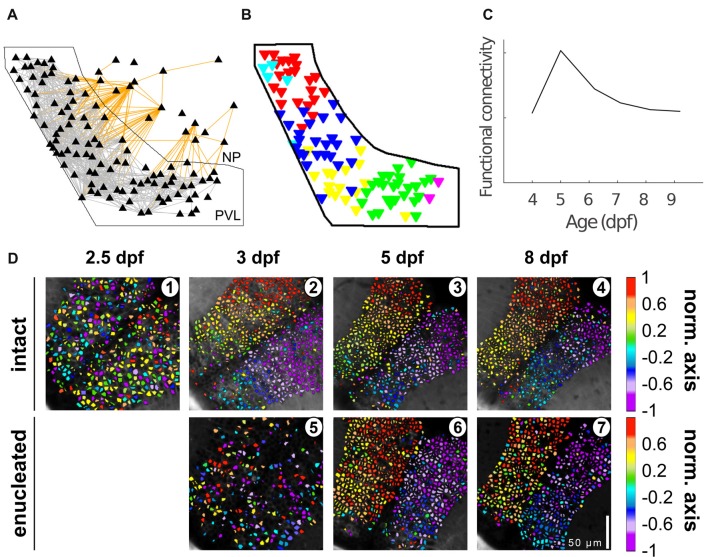
Tectal functional connectivity and neuronal assembly characteristics change over development. **(A)** Representation of the optic tectum as a graph. Each node (triangle) represents a neuron, and each edge represents correlation exceeding a threshold, i.e., functional connectivity between two neurons (gray lines for intra-PVL edges and orange lines for edges involving NP neurons). The solid black outline shows the boundary of the PVL. **(B)** Six communities (color coded) were detected in the graph presented in **(A)**. **(C)** Functional connectivity metrics peaked at 5 dpf and declined afterwards. **(D)** Examples of the spatial organization of the spontaneous activity along the caudo-rostral axis of an intact (1–4) and enucleated larva (5–7) at different developmental stages. Scale bar represents 100 mm. Color bar: position along the caudo-rostral axis (the neurons are color coded according to the centroid azimuthal position of the assembly to which they belong; reproduced with permission from Avitan et al., 2017; Pietri et al., 2017).

Avitan et al. (2017) also showed changes over development in the characteristics of tectal spontaneous activity at the single cell and population level (Figure 3). At 5–6 dpf both single-neuron event frequency and correlation at the population level increased while the dimensionality of the population activity decreased. These changes were then reversed by 8 dpf, showing similar values to those observed at 4 dpf. These authors studied the functional organization of the tectum by treating the tectum as a graph, a mathematical structure composed of a set of nodes (representing neurons) joined together in pairs by edges (representing pair-wise correlation; Rubinov and Sporns, 2010). These tectal graphs were studied using metrics whose changes over development revealed corresponding modifications in the functional connectivity reorganization, with most measures (graph mean degree, clustering coefficient and global efficiency) peaking at 5 dpf and declining afterwards. At all ages inspected (4–9 dpf) graph degree distribution followed a power law, a topological feature of a network often called “scale free,” which indicates a high proportion of neurons with low connectivity and low proportion of “hub” neurons with higher connectivity than expected. Thus, although the underlying topology of tectal functional networks remains robust, over development tectal functional connectivity substantially refines.

## Dependence of Tectal Functional Circuitry on Visual Experience

To assess the role of visual experience in the development of the tectal functional circuit, Pietri et al. (2017) studied the development of spontaneous activity in tecta deprived of visual inputs (bi-lateral enucleations performed at 54–58 hpf). Under these conditions, the amplitude, frequency and synchronization of calcium events showed a significant increase compared to the intact larvae. The excitatory retinal inputs that normally commence at 3 dpf therefore seem to play a major role in the maturation of tectal responses. The temporal structure (the pair-wise correlations between the activity of the neurons) was also significantly impacted, displaying lower correlation levels at 3 dpf with respect to intact larvae. Surprisingly, by 5 dpf, the significant pair-wise correlations in enucleated larvae increased by ~120% and remained at this level to 8 dpf. In contrast, the development of the spatial structure of the spontaneous activity was initially delayed, but by 8 dpf, when the tectal circuitry is functionally mature (Niell and Smith, 2005; Romano et al., 2015), it reached similar values to those of intact larvae.

Avitan et al. (2017) further tested the role of retinal activity (both evoked and spontaneous) in shaping the development of spontaneous activity, by performing bilateral and unilateral enucleation at 24 hpf and recording tectal spontaneous activity at 6 dpf. In the developing zebrafish, spontaneous retinal waves were observed during 2.5–3.5 dpf (Zhang et al., 2016) and hence in the enucleated larvae, the tectal circuit did not sense either spontaneous retinal waves nor evoked retinal input. Deprived tecta in the bilateral enucleation case and both the intact and deprived tecta in the unilateral enucleation case were examined. Neural assemblies were still present in the unilaterally and bilaterally deprived tecta, although they were slightly fewer in number and showed slightly less spatial organization, suggesting that their formation is at least partially driven endogenously. The correlation structure of intact tecta in the unilateral enucleation case was much more similar to the structure of the deprived tecta from both unilateral and bilateral enucleations than to the tecta of intact larvae at the same age, indicating a role for interhemispheric connections in transfer of information to the deprived tectum.

Besides enucleations, Avitan et al. (2017) also studied the role of visual input in shaping tectal spontaneous activity and instructing behavior by rearing the fish in the dark until 6 dpf, keeping spontaneous retinal activity intact while preventing visual experience (Figure 3). While measures at the single-neuron level such as event frequency were similar to the normally reared fish (Niell and Smith, 2005; Avitan et al., 2017), there were substantial changes in population level characteristics of spontaneous activity (such as correlations between pairs of neurons) of spontaneous activity, with a lower number of neuronal assemblies and reduced functional connectivity in dark-reared fish. This difference in the statistics of spontaneous activity was reflected in altered visually guided behavior, where dark-reared fish hunted fewer Paramecia compared to the normally-reared fish. This difference persisted also after exposing dark-reared fish to normal rearing conditions from 6 dpf to 9 dpf, suggesting that early visual experience has a long-lasting effect on visually guided behavior.

Together these findings support the hypotheses that: (i) neither visual experience nor spontaneous activity in the retina are essential for the establishment of spatially compact neural assemblies in the optic tectum; but (ii) retinal activity does play at least some role in shaping functional connectivity in the tectum. The emergence of spatial structure in spontaneous activity is likely determined by a pre-programmed mechanism that organizes the circuit architecture of the optic tectum, but activity-dependent mechanisms may further refine these spatial structures.

## The Role of the Tectal Spontaneous Activity in Motor Behavior

To investigate the functional role of tectal neuronal assemblies Romano et al. (2015) compared several assembly properties with behavioral characteristics during prey-capture behaviors, e.g., the relative position of the prey (Paramecia) and its angular size at the time of detection. They showed that the assemblies emerging in areas of the optic tectum that represent lateral positions of the visual field of the larva (caudal regions of the optic tectum), are composed of neurons with significantly narrower spatial tuning curves and higher activation levels than those situated in areas that represent rostral positions. Consistently, larvae detected prey more often when they were at the lateral positions of the visual field of the larva. The angular size of Paramecia at the moment of detection represented ~5° on the larva’s retina. Remarkably, neuronal groups induced by similarly-sized light spots were over-represented among the spontaneous neuronal assemblies.

In addition, it was found that the activation of some spatially compact assemblies was associated with brief episodes of tail movements. Notably, when the activation of a spontaneous assembly preceded a tail flip with kinematics that indicated a turning movement, the assembly corresponded to the contralateral tectal hemisphere in 93% of the cases. Thus, the spontaneous activations of tectal assemblies can be interpreted as a latent state that allows a better response to events that are biologically meaningful (e.g., visual stimuli resembling prey).

## Discussion and Perspectives

Overall, the studies discussed here show that the spontaneous activity of the optic tectum, far from being noise, presents activity patterns (neuronal assemblies) that emerge from the intrinsic dynamics of the tectal circuit. These assemblies mimic the spatial organization of visually induced responses associated with the larva’s prey, and are organized according to the tectum’s retinotopic map. Neither visual experience nor intrinsic retinal activity are essential for the basic functional development of the tectal network. This pre-programmed innate mechanism could be an advantageous strategy in species without parental care, enabling the prompt execution of vital behaviors soon after hatching, without requiring prior visual experience. However, visual experience is important for further fine tuning the tectal network. This hypothesis is supported by a recent study which also shows that neuronal assemblies could be generated via artificially induced Hebbian plasticity (Carrillo-Reid et al., 2016).

Tectal circuitry thus appears to be adapted for the biologically relevant statistics of the environment (expectations or priors). This salience of biologically relevant stimuli could be interpreted as a biased “attentional-like” process imposed by the circuit’s functional connectivity. In other words, the tectal circuitry is more sensitive to biologically relevant objects (e.g., prey) in comparison to objects that are less relevant for the survival of the larva. Moreover, the spontaneous activity structure reflects the tectal connectivity based on preferred local circuit states with attractor-like dynamics. This mechanism may help increase the signal-to-noise ratio for stimulus detection in low-contrast backgrounds, suggesting that the tectal network is wired for its functional role. Some assemblies are capable of predicting specific self-generated motor behaviors, potentially underlying internal decision making.

Performing similar experiments in others brain areas could shed light on the brain’s basic principles underlying information processing. In addition, future studies on zebrafish models for human neurological diseases (Best and Alderton, 2008; Sager et al., 2010), may contribute to the understanding of neuronal anomalies in functional connectivity underlying neurological disorders, such as Rett’s syndrome (Panier et al., 2013), or autism (Stewart et al., 2014).

## Author Contributions

EM, LA, GG and GS wrote the manuscript.

## Conflict of Interest Statement

The authors declare that the research was conducted in the absence of any commercial or financial relationships that could be construed as a potential conflict of interest.
